# 846. Trend Analysis of Cause-Specific Mortality among HIV-Infected Veterans: A 35-Year Study

**DOI:** 10.1093/ofid/ofab466.1041

**Published:** 2021-12-04

**Authors:** Kartavya J Vyas

**Affiliations:** Emory University, Decatur, Georgia

## Abstract

**Background:**

The aims are to estimate the rates for, and examine the trends of, all-cause and cause-specific mortality since the beginning of the epidemic, in an effort to better forecast future mortality patterns and potentially prevent premature death.

**Methods:**

All patients in the HIV Atlanta VA Cohort Study (HAVACS), an ongoing, open cohort of all HIV-infected veterans who ever sought or are seeking care at the Atlanta VA Medical Center, with a documented HIV diagnosis between January 1982 and December 2016 are included. All-cause and cause-specific mortality rates are calculated annually and for the study period, and age-adjusted to the 2000 U.S. standard population. Join-point regression analyses are performed to calculate annual percent changes (APC) and 95% CIs during periods of time when significant changes in trends are observed.

**Results:**

The analytic sample consisted of 4,674 patients; of whom 1,752 (36.8%) died. The age-adjusted all-cause mortality rate per 100 PY (95% CI) is 19.0 (9.9, 28.2); this rate decreased 45.2% annually from 1983 to 1987, and thereafter became relatively stable. The age-adjusted mortality rates for AIDS–opportunistic infection (aIR=19.0, 95% CI=17.0, 21.0), cardiovascular (aIR=16.2, 95% CI=9.2, 23.1; APC=-2.0), infection (aIR=20.7, 95% CI=10.3, 31.1), liver (aIR=13.8, 95% CI=9.7, 18.0; APC=-0.6), pulmonary (aIR=24.6, 95% CI=3.4, 45.8; APC=-0.3), renal (aIR=17.6, 95% CI=11.1, 24.1; APC=-1.3), and violence (aIR=14.7, 95% CI=9.2, 20.2; APC=-2.8) have all decreased since the beginning of the epidemic, most markedly for AIDS–opportunistic infection (APC=-18.0; 95% CI=-31.9, -1.4) and infection (APC=-3.4; 95% CI=-6.5, -0.3). In contrast, the age-adjusted mortality rates for AIDS–opportunistic malignancy (aIR=32.4, 95% CI=15.9, 48.9; APC=1.5), malignancy (aIR=13.2, 95% CI=6.2, 20.2; APC=1.1), and sudden death (aIR=9.6, 95% CI=6.1, 13.1; APC=32.2) have increased since the beginning of the epidemic.

Figure 1. Joinpoint regression analysis of age-adjusted mortality rates in the HAVACS cohort, 1982-2016 (n=4,674).

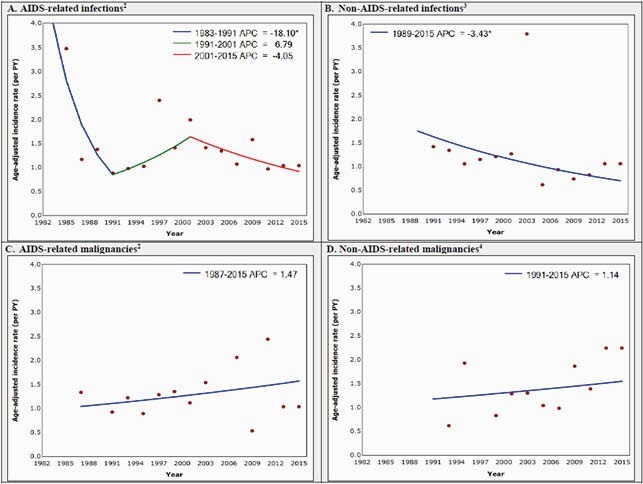

AIDS, acquired immune deficiency syndrome; APC, annual percent change; HAVACS, HIV Atlanta VA Cohort Study; HIV, human immunodeficiency virus; PY, person-years. *Statistically significant at α=0.05. 1. 2000 U.S. standard population; excludes deaths for which the date is unknown (n=46). 2. Coding Causes of Death in HIV (CoDe) protocol adapted to classify causes of death; AIDS-related illnesses refers to an appended list of AIDS-defining illnesses (1993 definition). 3. Pulmonary infections included in pulmonary, not infection. 4. Hepatocellular carcinoma included in liver, not malignancy.

**Conclusion:**

HIV-infected veterans are experiencing decreasing mortality rates due to almost all causes of death, principally infections; however, increasing mortality rates due to malignancies and sudden death are observed. Identifying risk factors for those causes on the rise may help realign resources and mitigate disease burden in this population.

**Disclosures:**

**All Authors**: No reported disclosures

